# Chloroplast-localized iron superoxide dismutases FSD2 and FSD3 are functionally distinct in *Arabidopsis*

**DOI:** 10.1371/journal.pone.0220078

**Published:** 2019-07-22

**Authors:** Daniel R. Gallie, Zhong Chen

**Affiliations:** Department of Biochemistry, University of California, Riverside, CA, United States of America; Medical College of Georgia at Augusta University, UNITED STATES

## Abstract

Superoxide dismutases (SODs) protect against reactive oxygen species (ROS) by detoxifying superoxide. Three types of SOD are present in plants: FeSOD, CuSOD, and MnSOD. The *Arabidopsis thaliana* genome contains three FeSOD genes, in which two (FSD2, and FSD3) are targeted to chloroplast thylakoids. Loss of FSD2 or FSD3 expression impairs growth and causes leaf bleaching. FSD2 and FSD3 form heterocomplexes present in chloroplast nucleoids, raising the question of whether FSD2 and FSD3 are functionally interchangeable. In this study, we examined how loss of FSD2 or FSD3 expression affects photosynthetic processes and whether overexpression of one compensates for loss of the other. Whereas loss of the cytosolic FSD1 had little effect, an *fsd2* mutant exhibited increased superoxide production, reduced chlorophyll levels, lower PSII efficiency, a lower rate of CO_2_ assimilation, but elevated non-photochemical quenching (NPQ). In contrast, *fsd3* mutants failed to survive beyond the seedling stage and overexpression of FSD2 could not rescue the seedlings. Overexpression of FSD3 in an *fsd2* mutant, however, partially reversed the *fsd2* mutant phenotype resulting in improved growth characteristics. Overexpression of FSD2 or FSD3, either individually or together, had little effect. These results indicate that, despite functioning as FeSODs, FSD2 and FSD3 are functionally distinct.

## Introduction

Although photosynthesis is critical to most plants to capture and convert absorbed light energy into chemical energy, an inevitable consequence of this process is the generation of reactive oxygen species (ROS). As ROS can be highly damaging, plants have evolved several means to manage ROS levels that include enzymatic and nonenzymatic mechanisms [1]. One such mechanism involves superoxide dismutases (SODs) which reduce superoxide to hydrogen peroxide (H_2_O_2_) that in turn is converted to water by catalase [2]. The three types of SOD present in plants based on their metal cofactors are iron SOD (FeSOD), copper–zinc SOD (Cu/ZnSOD) and manganese SOD (MnSOD) each located in specific cellular locations including the cytosol, mitochondria, and chloroplasts [3]. Three FeSOD genes (FE SUPEROXIDE DISMUTASE) are present in the genome of *Arabidopsis thaliana* (FSD1, FSD2, and FSD3) [4]. Although FSD1 has been suggested to localize to the chloroplast [5–8], the mitochondrial membrane [9], or the plasma membrane [10], more recent evidence suggests it is a cytosolic protein whereas FSD2 and FSD3 are targeted to the chloroplast [11].

Loss of FeSOD expression can affect photosynthetic functioning. In the photosynthetic cyanobacterium, *Synechococcus*, loss of the cytosolic FeSOD results in inactivation of PSI and increased photooxidative stress [12–14]. *Arabidopsis* mutants deficient in the chloroplast-localized FSD2 or FSD3 exhibit a pale green phenotype [11]. The combinatorial loss of FSD2 and FSD3 expression results in a more severe albino phenotype [11], suggesting that FSD2 and FSD3 function together to maintain photosynthetic health. Interestingly, loss of FSD1 expression had little effect on photosynthetic functioning or plant growth [11]. As superoxide is unable to cross phospholipid membranes, this suggests that the cytosolic location of FSD1 may prevent it from protecting the chloroplast from superoxide generated during photosynthesis. Photoprotection conferred by SOD activity is not limited to FSD2 and FSD3 as a reduction in the expression of CSD2, a chloroplast-localized Cu/ZnSOD [7], resulted in lower photosynthetic activity and abnormal chloroplast development characterized by a reduction in the quantity of granal thylakoids [15]. Overexpression of FSD2 or FSD3, either alone or together, had no effect on plant growth under normal growth conditions [11]. Although overexpression of FSD2 or FSD3 failed to provide increased tolerance to oxidative stress imposed by methyl viologen, overexpression of both did improve tolerance [11].

Expression of FSD3-GFP suggested localization to plastid nucleoids whereas FSD2-GFP was observed throughout the chloroplast stroma [11]. Nevertheless, interaction studies *in vitro* and *in vivo* suggest that FSD2 and FSD3 form a heterocomplex in plastid nucleoids [11]. Plastid-encoded plastid RNA polymerase (PEP) gene expression was specifically impaired in *fsd2* and *fsd3* mutants while nucleus-encoded plastid RNA polymerase (NEP) gene expression was actually higher in the mutants than in wild-type plants [11]. As mutants of components of transcriptionally active chromosomes (TACs) from Arabidopsis and mustard exhibited expression patterns of plastid-encoded genes that were similar to *fsd2* and *fsd3* mutants [16], the FSD2-FSD3 heterodimer has been suggested to function as part of the PEP complex during plastid gene expression in plastid nucleoids to protect TACs against ROS during early chloroplast development [11]. Supporting this, electrospray ionization ion-trap tandem mass spectrometry analysis identified FSD2 and FSD3 as TAC components [16] and the mustard FSD3 homolog co-purified with soluble RNA polymerase (sRNAP) [17].

As FSD2 and FSD3 form heterocomplexes in chloroplast nucleoids and the loss of either results in similar phenotypes, the extent to which FSD2 and FSD3 are functionally distinct remains unknown. In this study, we examined whether overexpression of FSD3 could compensate for loss of FSD2 expression or overexpression of FSD2 could compensate for loss of FSD3 expression. We observed that overexpression of FSD2 failed to rescue *fsd3* seedlings beyond the seedling stage. In contrast, overexpression of FSD3 in an *fsd2-1* mutant partially reversed its poor growth and low chlorophyll content. Overexpression of FSD3 partially reversed the elevated production of superoxide in *fsd2-1* mutant plants and partially reversed the low PSII efficiency and low rate of CO_2_ assimilation of the mutant while reducing the elevated non-photochemical quenching (NPQ) characteristic of *fsd2-1* mutant plants. These results indicate that, despite their presence as heterocomplexes in chloroplast nucleoids and the partial reversal of *fsd2-1* phenotypes by FSD3 overexpression, FSD2 and FSD3 exhibit functional differences.

## Materials and methods

### Plant growth

After surface-sterilization and cold treatment at 4°C for 4 days in the dark, *Arabidopsis* seeds were planted on 0.25 x MS agar plates containing 1% sucrose and grown at 20°C in a plant growth room supplemented with Sylvania Gro-Lite fluorescent bulbs (Sylvania, Danvers MA, USA) at a photon flux density (PFD) of 100 μmol photons m^-2^ s^-1^. For adult plants, seeds were germinated on MS agar plates containing 1% sucrose for 1 week prior to transfer to soil.

### Superoxide assay

The rate of superoxide production was measured spectrophotometrically as described [18, 19]. Arabidopsis seedlings were infiltrated with 10 ml of 10 mM citrate buffer pH 7.8 containing 50 μM XTT, i.e., sodium 3’-[1-(phenylamino)-carbonyl-3,4-triazolium]-bis(4-methoxy-6-nitro) benzenesulfonic acid hydrate, and exposed to 1900 PFD under constant temperature. The rate of superoxide production in the leaf samples was monitored spectrophotometrically every 15 min at 470 nm (extinction coefficient of 2.16 x 10^4^ M^-1^ cm^-1^) for 2 hr.

### Chlorophyll pigment measurements

Chlorophyll a and b were measured spectrophotometrically as described [20]. Leaf samples were ground in liquid nitrogen and extracted with 90% (v/v) acetone. The absorbance at 664 and 647 nm was determined and used to calculated chlorophyll a and b content by the equations: Chl a = 11.93A_664_-1.93A_647_ and Chl b = 20.36A_647_-5.50A_664_, respectively. Each experiment was repeated 2–3 times and representative results presented.

### Gas exchange and fluorescence measurements

Gas exchange and fluorescence measurements were performed using a LI-COR Li-6400 portable photosynthesis system (LI-COR, Lincoln, NE) with LI-6400-40 leaf chamber, a relative humidity of 50%, and ambient level of CO_2_. Fluorescence measurements were taken using overnight dark-adapted leaves. At the start of each experiment, the leaf was exposed to 2 min of far-red illumination (1 PFD) for the determination of F_o_ (minimum fluorescence in the dark-adapted state). Saturating pulses (0.8 s of 5000 PFD) were applied to determine the F_m_ or F_m_’ values. Actinic light, consisting of 90% of red light (λ = 630 ± 20 nm) and 10% blue light (λ = 470 ± 20 nm) was provided by LED (light emission diode) sources. F_s_ is the steady fluorescence yield during actinic illumination. F_o_’ (minimum fluorescence in the light-adapted state) was determined in the presence of far-red (λ = 740 nm) light after switching off the actinic light. A total of four to six samples were measured in each experiment. All data presented were calculated from at least three independent measurements. Conventional fluorescence nomenclature was used [21]. NPQ was calculated from (F_m_-F_m_’)/F_m_’ and φPSII from (F_m_’-F_s_’)/F_m_’. NPQ_f_ and NPQ_s_ were determined as described [22].

### Statistical analysis

For each experiment, the mean and standard error reported of 4 to 6 replicates as indicated. Each mean value was subjected to Student’s *t-*test and values of *P* ≤ 0.05 were taken as statistically significant.

## Results

### Loss of FSD2 expression results in reduced photosynthetic activity

**Fig 1:**
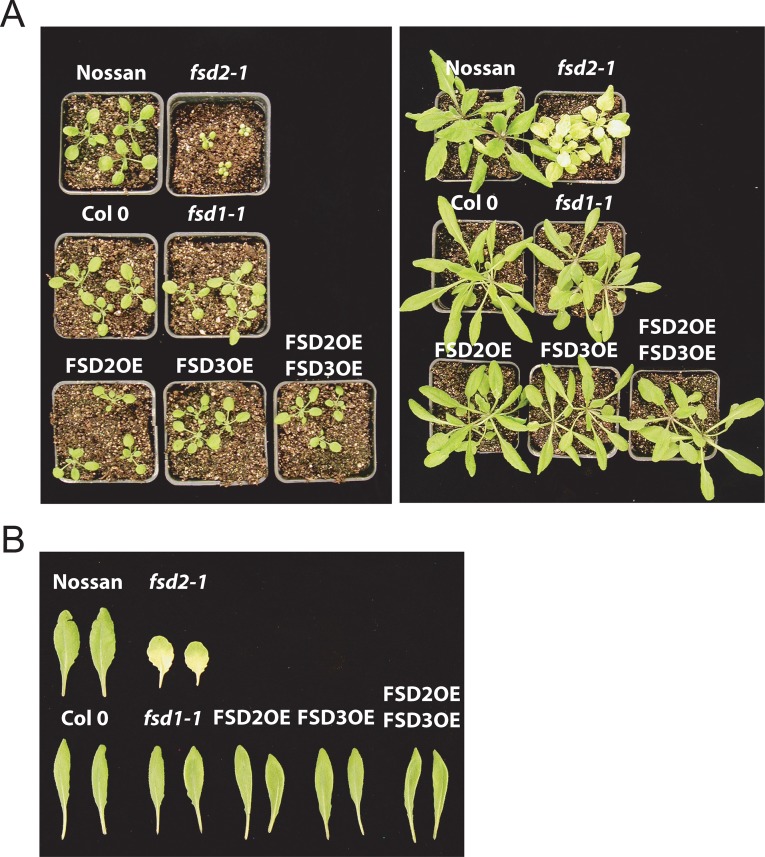
Growth phenotypes of FeSOD mutants. Plants were grown at 100 PFD to a similar developmental stage. In A, plants were grown at 100 PFD for 2.5 weeks (left panel) or 4 weeks (right panel). In B, leaves of each line from 4 week old adult plants grown at 100 PFD.

As reported previously [11], the absence of FSD2 expression in the *fsd2-1* mutant results in smaller plant stature due to slower growth under 100 PFD (Fig 1). Under these growth conditions, *fsd3* mutants such as *fsd3-1* and *fsd3-2* germinated but were severely bleached and died at the seedling stage whether grown in soil or on agar under conditions ranging from low to normal light. In agreement with previous work, loss of FSD1 expression had little effect on growth (Fig 1) [11]. Moreover, overexpression of FSD2 or FSD3, either individually or together, had little effect on plant growth or size (Fig 1), suggesting that increasing FSD expression provided little benefit under these growth conditions.

To examine whether loss of FSD expression resulted in a reduced capacity to scavenge ROS, superoxide production was measured in leaves of adult plants grown under 100 PFD. Although superoxide production was lower in Nossen (the genetic background for the *fsd2* mutant) than in Col 0 (the genetic background for the *fsd1* and *fsd3* mutants), the level of superoxide production was substantially higher in the *fsd2-1* mutant relative to Nossen (Table 1). Superoxide production increased slightly in leaves of *fsd1-1* plants relative to the levels in Col 0 leaves but this difference was not significant (Table 1). Superoxide production was measured also in plants overexpressing in either FSD2 or FSD3. Previous work had shown that overexpression of FSD2 or FSD3 in Col 0 using the CaMV 35S promoter successfully complemented the *fsd2-1* or *fsd3-1* mutants, respectively [11], demonstrating that each was expressed. Using these same lines, we examined if overexpression of FSD2 and/or FSD3 would provide any benefit to wild-type plants under the conditions employed. Overexpression of FSD2 or FSD3, however, had little effect on superoxide production in adult plants (Table 1). A slight increase in superoxide production was observed in leaves of plants overexpressing FSD2 and FSD3 but this difference was not significant.

**Table 1:** Loss of *fsd2* results in an increase in superoxide.

	Superoxide production^a^ (nmol/min/g FW)	t-test
**Nossen**	5.82 ± 1.22	
***fsd2-1***	17.5 ± 2.34	P<0.05
**Col 0**	8.22 ± 1.63	
***fsd1-1***	11.0 ± 1.83	P = 0.190
**FSD2OE**	9.09 ± 1.70	P = 0.628
**FSD3OE**	8.84 ± 1.43	P = 0.700
**FSD2OE/FSD3OE**	10.6 ± 1.81	P = 0.248

^a^Determined from three replicates of three-week old plants (4.5 weeks for *fsd2-1*) grown at 100 PFD.

The *fsd2-1* mutant not only grew slower but was noticeably less green (Fig 1) as reported previously [11]. This was confirmed by quantitation of chlorophyll levels in which chlorophyll a (Chl a) and chlorophyll b (Chl b) levels were substantially lower in the *fsd2-1* mutant relative to wild-type Nossen plants (Table 2). The extent of reduction of Chl a and Chl b levels was similar resulting in a Chl a/b ratio close to that observed in Nossen. In contrast to the *fsd2-1* mutant, the chlorophyll levels in *fsd1-1* were largely unchanged from those in Col 0 (Table 2). Overexpression of FSD2 or FSD3, either individually or together, did not alter chlorophyll levels or the Chl a/b ratio.

**Table 2:** *fsd2* mutant plants exhibit reduced chlorophyll content.

	Chl a^a^ (μg/g FW)	Chl b^a^ (μg/g FW)	Chl a+b^a^ (μg/g FW)	Chl a/b^a^
**Nossen**	634 ± 15.9	217 ± 9.4	851 ± 24.4	2.93 ± 0.08
***fsd2-1***	478 ± 24.8	159 ± 3.1	636 ± 27.6	3.01 ± 0.10
**Col 0**	687 ± 19.6	235 ± 0.89	922 ± 19.8	2.92 ± 0.08
***fsd1-1***	664 ± 21.4	224 ± 14.5	888 ± 32.5	2.98 ± 0.15
**FSD2OE**	662 ± 28.0	216 ± 9.2	878 ± 36.3	3.07 ± 0.06
**FSD3OE**	679 ± 27.0	222 ± 11.6	901 ± 38.5	3.06 ± 0.04
**FSD2OE/FSD3OE**	678 ± 22.0	227 ± 3.6	904 ± 25.5	2.99 ± 0.05

^a^Determined from three replicates of two-week old plants (3 weeks for *fsd2-1*) grown at 100 PFD.

The reduction in chlorophyll levels and the slower growth of the *fsd2-1* mutant may indicate altered photosynthetic activity. To examine this, the rate of CO_2_ assimilation was measured in adult leaves of the *fsd2-1* mutant and the other lines. At 100 PFD, the light level used for plant growth, the rate of CO_2_ assimilation in *fsd2-1* was reduced to approximately 25% of that observed in Nossen (Table 3). The reduction in the rate of CO_2_ assimilation was not due to a reduction in the internal concentration of CO_2_ (C_i_) in *fsd2-1* as C_i_ was actually higher in *fsd2-1* than in Nossen (Table 3). Although the rate of CO_2_ assimilation was higher when leaves were exposed to 200 PFD, the rate in *fsd2-1* remained approximately 25% of that observed in Nossen (Table 3). In contrast, the rate of CO_2_ assimilation and C_i_ in *fsd1-1* were not significantly different from those in wild-type Col 0 at either light level (Table 3). Overexpression of FSD2 or FSD3, either individually or together, had little to no effect on the rate of CO_2_ assimilation. These results suggest that the slow growth phenotype of the *fsd2-1* mutant may be a result of its substantially lower photosynthetic activity.

**Table 3:** Loss of *fsd2* results in a decreased rate of CO_2_ assimilation.

	CO_2_ assimilation (μmol CO_2_/m^2^/sec/g FW)^a^			
	100 PFD	Ci (ppm)	200 PFD	Ci (ppm)
**Nossen**	3.96 ± 0.63	290 ± 11.7	5.66 ± 0.38	299 ± 15.1
***fsd2-1***	0.94 ± 0.30	381 ± 13.0	1.45 ± 0.27	365 ± 25.4
**Col 0**	4.12 ± 0.58	296 ± 20.3	5.88 ± 0.38	306 ± 12.8
***fsd1-1***	3.93 ± 0.44	304 ± 31.4	5.73 ± 0.41	299 ± 30.1
**FSD2OE**	3.85 ± 0.41	293 ± 37.0	5.55 ± 0.44	334 ± 7.6
**FSD3OE**	3.84 ± 0.39	293 ± 20.1	5.83 ± 0.51	296 ± 14.4
**FSD2OE/FSD3OE**	3.62 ± 0.36	319 ± 10.6	5.54 ± 0.21	325 ± 13.8

^a^Determined from three replicates of three-week old plants (4.5 weeks for *fsd2-1*) grown at 100 PFD.

### Loss of FSD2 expression results in an increase in NPQ

**Fig 2:**
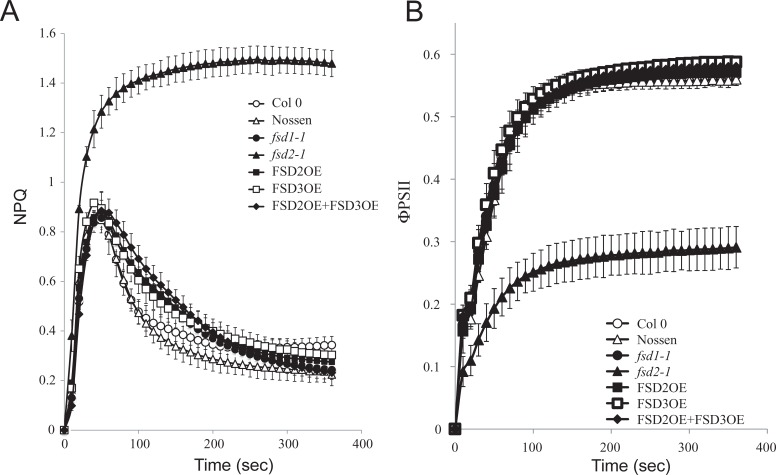
The *fsd2-1* mutant exhibits increased induction of non-photochemical quenching under low light. (A) NPQ and (B) PSII efficiency (ΦPSII) were simultaneously measured in dark-adapted Col 0 (open circles), Nossen (open triangles), *fsd1-1* (filled circles), *fsd2-1* (filled triangles), FSD2OE (filled squares), FSD3OE (open squares), and FSD2OE and FSD3OE (filled diamonds) plants grown at 100 PFD to a similar developmental stage and exposed to 111 PFD. Six replicates were assayed for each line with the average and standard error reported.

NPQ and photosynthesis contribute to the quenching of absorbed light energy and as such are competing quenching processes. As the *fsd2-1* mutant exhibits reduced photosynthetic activity, NPQ was measured to determine whether it exhibits an altered induction profile. Dark-adapted leaves from plants grown at 100 PFD that were exposed to 111 PFD exhibited an initial rapid induction of total NPQ followed by a partial relaxation upon the induction of photosynthetic activity (Fig 2A). Although the NPQ induction profile in *fsd1-1* leaves was similar to that in Col 0, NPQ in *fsd2-1* leaves was induced to a substantially higher level during exposure to light and did not relax as it did in Nossen (Fig 2A). The higher level of NPQ in *fsd2-1* correlated with a substantially lower level of PSII efficiency (ΦPSII) relative to Nossen (Fig 2B). In contrast, ΦPSII in *fsd1-1* was similar to the level observed in Col 0 (Fig 2B).

**Fig 3:**
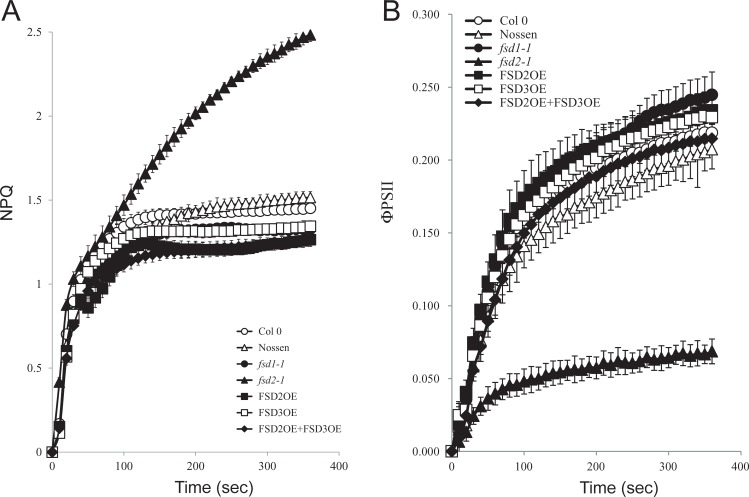
The *fsd2-1* mutant exhibits increased induction of non-photochemical quenching under high light. (A) NPQ and (B) PSII efficiency (ΦPSII) were simultaneously measured in dark-adapted Col 0 (open circles), Nossen (open triangles), *fsd1-1* (filled circles), *fsd2-1* (filled triangles), FSD2OE (filled squares), FSD3OE (open squares), and FSD2OE and FSD3OE (filled diamonds) plants grown at 100 PFD to a similar developmental stage and exposed to 400 PFD. Five replicates were assayed for each line with the average and standard error reported.

When dark-adapted leaves from plants grown at 100 PFD were exposed to 400 PFD, an initial rapid induction of total NPQ was observed for all lines followed by a plateau for the duration of the measuring time (Fig 3A). The only exception was *fsd2-1* in which NPQ was induced similar to the other lines but continued to increase while the NPQ levels in the other lines began to level off (Fig 3A). The ΦPSII in *fsd2-1* was substantially lower than in the other lines, including *fsd1-1* (Fig 3B).

**Fig 4:**
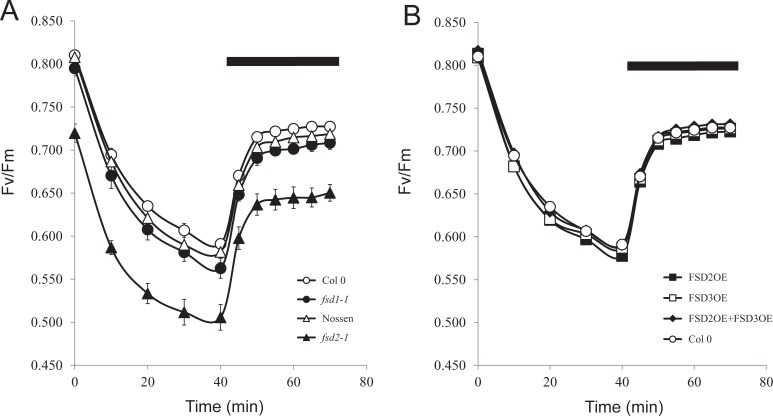
FSD2 is required to protect against photodamage to PSII. Leaves from adult plants grown at 100 PFD to a similar developmental stage were exposed to 1200 PFD for 40 min after which the leaves were transferred to darkness. Fv/Fm was monitored during light exposure and following transfer to darkness. The black bar in each panel indicates the period of dark treatment. (A) Col 0 (open circles), Nossen (open triangles), *fsd1-1* (filled circles), *fsd2-1* (filled triangles). (B) Col 0 (open circles), FSD2OE (filled squares), FSD3OE (open squares), and FSD2OE and FSD3OE (filled diamonds). Four replicates were assayed for each line with the average and standard error reported.

To examine whether exposure to light affected the quantum yield in *fsd2-1* leaves to a greater extent than in wild-type plants, the fluorescence parameters Fv and Fm were measured and the Fv/Fm ratio calculated. Dark-adapted leaves from plants grown at 100 PFD that were exposed to high light (1200 PFD) exhibited a rapid decrease in Fv/Fm that rose upon transfer to dark (Fig 4A). The maximum quantum yield was lower in *fsd2-1* and decreased to a greater extent than in Nossen (Fig 4A). In contrast, the quantum yield in *fsd1-1* decreased only slightly relative to that observed in Col 0 (Fig 4A). No significant change in Fv/Fm was observed in plants overexpressing FSD2 or FSD3, either individually or combinatorically (Fig 4B). The reduction in maximum quantum yield in dark-adapted *fsd2-1* leaves indicates a level of altered function of PSII reaction centers not present in wild-type plants.

NPQ is composed of qE which dissipates excess absorbed excitation energy as heat, state transition quenching (qT), photoinhibitory processes (qI), and qZ, which is distinct from qT [23]. These processes contributing to total NPQ can be grouped into those that relax quickly or slowly following transfer to dark and can be measured as fast (NPQ_f_) and slow (NPQ_s_) relaxation components of NPQ during recovery from exposure to high light. NPQ_f_ largely represents qE whereas NPQ_s_ represents slower components of NPQ, including qI. If the induction of NPQ in *fsd2-1* leaves observed in Figs 2A and 3A is indicative of an increase in qE, this would be reflected in an elevated NPQ_f_ whereas an increase in photodamage in *fsd2-1* as suggested in Fig 4A would be reflected in an increase in NPQ_s_. NPQ_f_ was significantly elevated in *fsd2-1* leaves whereas NPQ_s_ was only slightly increased, but not significantly, over that observed in Nossen (Table 4). This suggests that under these conditions the elevated total NPQ observed in *fsd2-1* is largely due to fast relaxation components of NPQ such as qE although some degree of photoinhibition may have occurred during the measuring period as well. Neither NPQ_f_ nor NPQ_s_ was significantly altered in *fsd1-1* relative to Col 0 (Table 4), consistent with an NPQ induction profile that is similar to that in Col 0 (Fig 2A). Overexpression of FSD2 or FSD3, either individually or together, had little to no effect on either NPQ_f_ or NPQ_s_.

**Table 4:** *fsd2* mutant plants exhibit elevated NPQ_f_ and NPQ_s_ relaxation.

	NPQ_f_^a^	NPQ_s_^a^
**Nossen**	0.117 ± 0.021	0.182 ± 0.018
***fsd2-1***	0.998 ± 0.21	0.203 ± 0.036
**Col 0**	0.128 ± 0.044	0.169 ± 0.034
***fsd1-1***	0.097 ± 0.034	0.163 ± 0.052
**FSD2OE**	0.106 ± 0.034	0.174 ± 0.027
**FSD3OE**	0.111 ± 0.035	0.185 ± 0.023
**FSD2OE/FSD3OE**	0.119 ± 0.032	0.177 ± 0.010

^a^Determined from three replicates of two-week old plants (3 weeks for *fsd2-1*) grown at 100 PFD that were exposed to 1000 PFD for 25 min prior to relaxation in dark.

**Fig 5:**
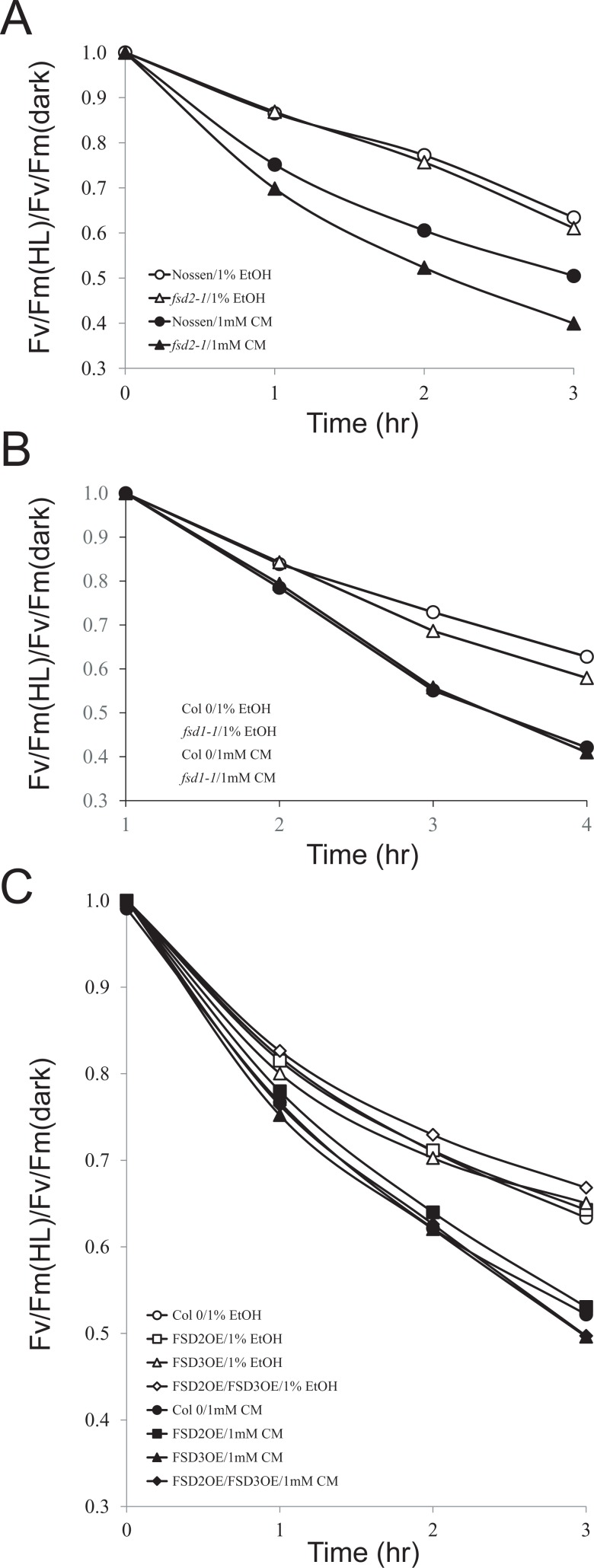
FSD2 is required to protect against photoinhibition. Leaves from adult plants grown at 100 PFD to a similar developmental stage were exposed to light at 500 PFD for the times indicated after vacuum infiltration in the presence of 1 mM chloramphenicol (Cm) or in ethanol carrier only (EtOH). Photoinhibition is reported as the ratio of Fv/Fm during high light treatment to the Fv/Fm in dark adapted leaves prior to light exposure. (A) Nossen and *fsd2-1*, (B) Col 0 and *fsd1-1*, and (C) Col 0, FSD2OE, FSD3OE, and FSD2OE/FSD3OE.

Photoinhibition can involve damage to PSII reaction centers as a means to limit ROS generation and damaged PSII reaction centers require new protein synthesis for their repair [24–28]. Inhibiting chloroplast protein synthesis inhibits PSII repair and results in a greater reduction in the quantum efficiency (i.e., Fv/Fm) during light exposure [29]. To examine whether photoinhibition in *fsd2-1* leaves occurs at a greater level than in wild-type leaves in the absence of repair activity, adult leaves from plants grown under 100 PFD were infiltrated with either 1 mM chloramphenicol/1% ethanol to inhibit chloroplast protein synthesis or 1% ethanol alone and the leaves were then exposed to high light (500 PFD). When new protein synthesis was not inhibited, the quantum yield in *fsd2-1* leaves decreased at a rate similar to that observed in Nossen (Fig 5A). Although the quantum yield in Nossen decreased more rapidly when new protein synthesis was inhibited, it decreased even more rapidly in *fsd2-1*, suggesting photodamage to PSII. The quantum yield in *fsd1-1* decreased at a rate similar to that observed in Col 0 regardless of whether chloroplast protein synthesis was inhibited or not (Fig 5B), suggesting that *fsd1-1* does not experience greater photodamage as a result of the loss of FSD1 expression. Similar results were observed in plants overexpressing FSD2 or FSD3, either individually or together (Fig 5C), suggesting that an increase in FSD2 and/or FSD3 does not confer greater photoprotection.

### FSD3 overexpression can partially complement the loss of FSD2 expression

FSD2 and FSD3 were reported to localize to chloroplast thylakoids and not the stroma [11]. As each protein lacks a transmembrane domain, they were predicted to be attached to the stromal side of thylakoid membranes. Because of their association in heterocomplexes and co-localization in within chloroplast nucleoids [11], we examined whether overexpression of FSD3 (FSD3OE) could compensate for loss of FSD2 expression or whether overexpression of FSD2 (FSD2OE) could compensate for loss of FSD3 expression. Using the same lines in which FSD2 and/or FSD3 were overexpressed from the 35S promoter [11], we examined whether any level of cross-complementation could be achieved following their overexpression. FSD3OE plants were crossed with *fsd2-1* plants and homozygous *fsd2-1*/FSD3OE progeny isolated in subsequent generations. Similarly, FSD2OE plants were crossed with FSD3/*fsd3-1* heterozygous plants (as *fsd3-1* null plants were unable to grow to maturity) and progeny subsequently screened for *fsd3-1*/FSD2OE individuals.

**Fig 6:**
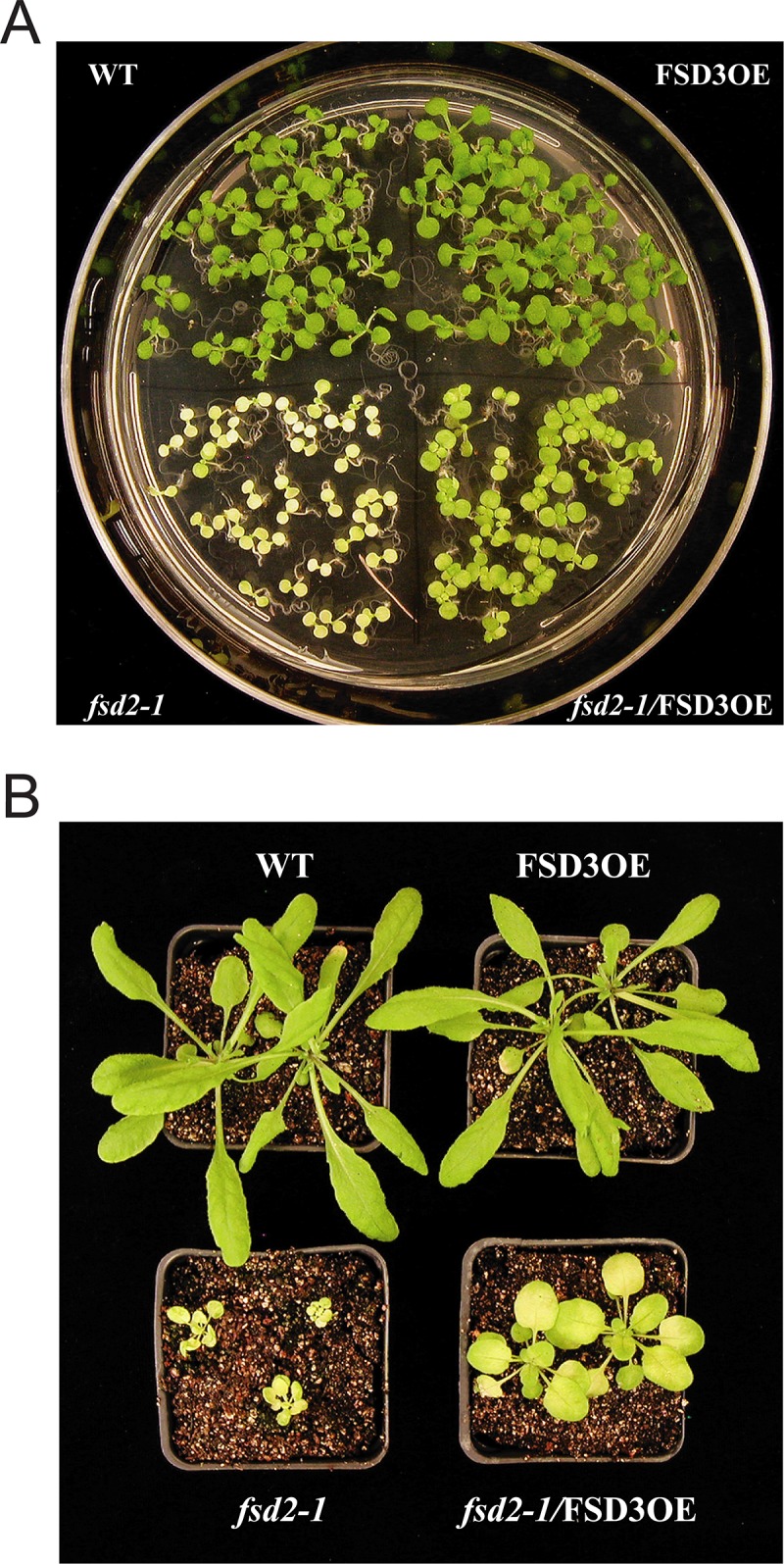
Overexpression of FSD3 partially complements the growth defects of the *fsd2-1* mutant. (A) Col 0, FSD3OE, *fsd2-1*, and *fsd2-1*/FSD3OE seed were germinated on MS medium and grown at 100 PFD for 7 days. (B) Col 0, FSD3OE, *fsd2-1*, and *fsd2-1*/FSD3OE plants were grown for 4 weeks in soil at 100 PFD.

No homozygous *fsd3-1*/FSD2OE progeny from FSD2OE plants that were heterozygous for FSD3 (i.e., FSD3/*fsd3-1*) could be isolated beyond the seedling stage, suggesting that overexpression of FSD2 was unable to compensate for loss of FSD3 expression in the *fsd3-1* mutant. Similar results were obtained when the *fsd3-2* mutant was used. In contrast, *fsd2-1*/FSD3OE progeny could be isolated. The cotyledons of *fsd2-1*/FSD3OE seedlings were greener than those of *fsd2-1* plants (Fig 6A) and adult *fsd2-1*/FSD3OE plants were larger and greener than *fsd2-1* plants although they remained smaller than Col 0 or FSD3OE plants (Fig 6B). These data suggest that overexpression of FSD3 can partially compensate for loss of FSD2 expression in the *fsd2-1* mutant to result in improved growth that is observed as early as the seedling stage and persists through to the adult stage of growth. The fact that overexpression of FSD3 could not fully reverse the poor growth phenotype of the *fsd2-1* mutant, however, suggests that FSD2 and FSD3 are not functionally identical.

Because *fsd2-1* leaves exhibit extensive bleaching with some green regions, particularly near leaf margins, whereas *fsd2-1*/FSD3OE leaves appear greener (Fig 6), the chlorophyll levels in each were measured quantitatively. As observed in leaves of adult plants, the levels of Chl a and Chl b in 7 day old plants were substantially lower in *fsd2-1* relative to Col 0 or FSD3OE (Table 5). The levels of Chl a and Chl b in *fsd2-1*/FSD3OE plants, however, were substantially higher than those in *fsd2-1*, although they remained lower than those in Col 0 or FSD3OE (Table 5). Interestingly in these younger plants, the Chl a/b ratio was lower in *fsd2-1* than in WT because the level of Chl a was disproportionately lower than the level of Chl b. However, the Chl a/b ratio in *fsd2-1*/FSD3OE was near to the ratio observed for Col 0 and FSD3OE. These data suggest that overexpression of FSD3 partially reverses the defects in chlorophyll levels observed in the *fsd2-1* mutant.

**Table 5:** The reduced chlorophyll content in *fsd2* mutant plants can be partially reversed by FSD3 overexpression.

	Chl a^a^ (μg/g FW)	Chl b^a^ (μg/g FW)	Chl a+b^a^ (μg/g FW)	Chl a/b^a^
**Col 0**	221 ± 3.19	84.9 ± 1.75	306 ± 13.8	2.60 ± 0.07
**FSD3OE**	223 ± 8.5	87.9 ± 2.84	310 ± 11.2	2.57 ± 0.04
***fsd2-1***	31.7 ± 0.40	16.9 ± 2.54	48.6 ± 2.85	1.91 ± 0.24
***fsd2-1* + FSD3OE**	106 ± 1.84	43.0 ± 1.28	149 ± 5.92	2.48 ± 0.07

^a^Determined from three replicates of 7-day old plants grown at 100 PFD.

The *fsd2-1* mutant was characterized by increased superoxide production relative to Nossen (Table 1). To examine whether overexpression of FSD3 in the *fsd2-1* mutant could reverse this elevated production of superoxide, superoxide was measured in leaves of 7 day plants grown under 100 PFD. Superoxide production was lower in the *fsd2-1* mutant overexpressing FSD3 relative to the level in the *fsd2-1* mutant itself although it remained higher than in WT (Table 6). Overexpression of FSD3 did not significantly alter the level of superoxide production relative to WT (Table 6). These data support the notion that overexpression of FSD3 partially compensates for loss of FSD2 expression in the *fsd2-1* mutant but fails to fully complement loss of FSD2 expression.

**Table 6:** The increased superoxide production in *fsd2* mutant plants can be partially reversed by FSD3 overexpression.

	Superoxide production^a^ (nmol/min/g FW)	t-test
**Col 0**	794 ± 163	
**FSD3OE**	1001 ± 292	P = 0.204
***fsd2-1***	2975 ± 820	P<0.05
***fsd2-1* + FSD3OE**	1435 ± 284	P<0.01

^a^Determined from three replicates of 7-day old plants grown at 100 PFD.

The maximum quantum yield was measured in *fsd2-1*/FSD3OE leaves to determine whether it correlated with the reduction in superoxide production observed for this line relative to *fsd2*. As observed above (Fig 4A), the maximum quantum yield in *fsd2* was reduced relative to WT (Table 7). The maximum quantum yield in *fsd2-1*/FSD3OE leaves, however, was substantially higher than that in *fsd2-1*, although it remained lower than in Col 0 or FSD3OE (Table 7). These data suggest that overexpression of FSD3 partially reverses the defect in the maximum quantum yield observed in the *fsd2-1* mutant.

**Table 7:** The reduction in the maximum quantum yield of *fsd2* mutant seedlings is partially reversed by FSD3 overexpression.

	Fv/Fm^a^ (Fm-Fo)/Fm	t-test
**Col 0**	0.766 ± 0.002	
**FSD3OE**	0.755 ± 0.007	P = 0.681
***fsd2-1***	0.110 ± 0.010	P<0.001
***fsd2-1* + FSD3OE**	0.536 ± 0.013	P<0.005

^a^Determined from three replicates of 10-day old seedlings grown at 100 PFD.

**Fig 7:**
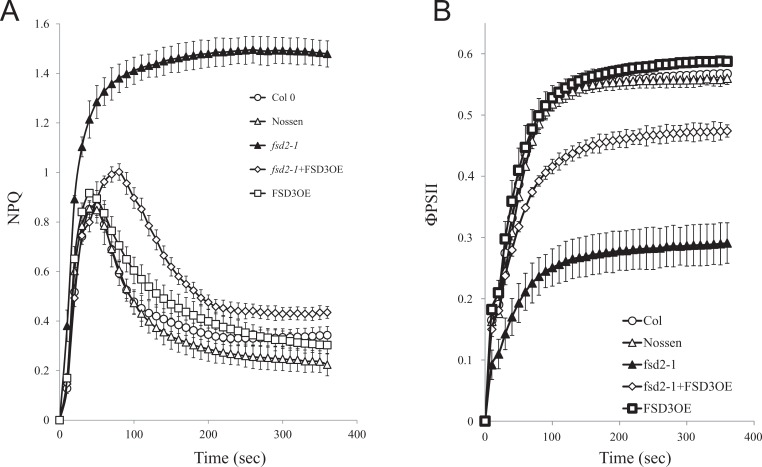
Overexpression of FSD3 partially complements the defects in NPQ and ΦPSII observed for the *fsd2-1* mutant under low light. (A) NPQ and (B) PSII efficiency (ΦPSII) were simultaneously measured in dark-adapted adult leaves of Col 0 (open circles), Nossen (open triangles), *fsd2-1* (filled triangles), *fsd2-1*/FSD3OE (open diamonds), and FSD3OE (open squares) plants grown at 100 PFD to a similar developmental stage and exposed to 111 PFD. Four replicates were assayed for each line with the average and standard error reported.

To examine the extent to which overexpression of FSD3 can restore a normal NPQ induction profile to the *fsd2-1* mutant, the induction of NPQ was measured in adult leaves. Dark-adapted leaves from plants grown at 100 PFD exhibited a transient induction of NPQ when exposed to 111 PFD that decreased as photosynthesis was activated (Fig 7A). The level of NPQ in Col 0, Nossen, and FSD3OE decreased to a similar low level following the initial transient induction. The level of NPQ in *fsd2-1* remained elevated at a substantially higher level throughout the period of measurement (Fig 7A). Following the initial transient induction, however, the level of NPQ in *fsd2-1*/FSD3OE leaves decreased to a level that was substantially lower than in *fsd2-1* and just moderately higher than that observed in Col 0, Nossen, and FSD3OE. Whereas the level of ΦPSII was induced to a substantially lower level in *fsd2-1* than in Col 0, Nossen, and FSD3OE, ΦPSII in *fsd2-1*/FSD3OE leaves was induced to level that was much closer, albeit slightly lower, than that in Col 0, Nossen, and FSD3OE (Fig 7B).

**Fig 8:**
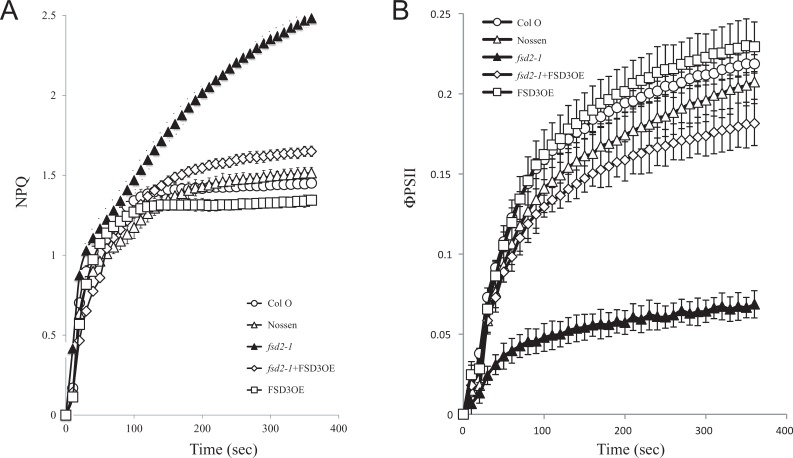
Overexpression of FSD3 partially complements the defects in NPQ and ΦPSII observed for the *fsd2-1* mutant under high light. (A) NPQ and (B) PSII efficiency (ΦPSII) were simultaneously measured in dark-adapted adult leaves of Col 0 (open circles), Nossen (open triangles), *fsd2-1* (filled triangles), *fsd2-1*/FSD3OE (open diamonds), and FSD3OE (open squares) plants grown at 100 PFD to a similar developmental stage and exposed to 400 PFD. Three replicates were assayed for each line with the average and standard error reported.

Similar results were obtained when a higher level of light was used. NPQ rose rapidly following exposure to 400 PFD. Although the level of NPQ in Col 0, Nossen, and FSD3OE reached a near plateau, NPQ continued to increase in *fsd2-1* to a substantially higher level (Fig 8A). In contrast, the level of NPQ in *fsd2-1*/FSD3OE leaves was substantially lower than in *fsd2-1* but modestly higher than that observed in Col 0, Nossen, and FSD3OE. The level of ΦPSII reached following exposure to 400 PFD was highest in FSD3OE and Col 0 whereas the level of ΦPSII was extremely low for the *fsd2-1* mutant relative to Nossen (Fig 8B). In *fsd2-1*/FSD3OE leaves, however, the level of ΦPSII was only moderately lower than in either Nossen or Col 0 and substantially higher than that in *fsd2-1* (Fig 8B). These results indicate that overexpression of FSD3 partially complements the defects in NPQ and ΦPSII observed for the *fsd2-1* mutant.

## Discussion

Of the three FeSOD genes present in *Arabidopsis*, FSD2 and FSD3 contribute substantially to chloroplast development and growth under normal conditions [11]. Interestingly, these two FeSODs are encoded by nuclear genes but are targeted to the chloroplast thylakoid membrane [11]. The third FeSOD member, FSD1, although previously suggested to localize to the chloroplast [5–8], was later shown to be cytosolic [11] and loss of FSD1 expression had little effect on photosynthetic processes. In contrast, loss of FSD2 or FSD3 expression resulted in severe leaf bleaching, slow growth, and delayed flowering. Loss of FSD3 expression appears to have a more profound effect on plant growth than does loss of FSD2 expression [11]. Indeed, *fsd3-1* and *fsd3-2* mutants failed to survive beyond the seedling stage under the growth conditions employed in this study whereas *fsd2-1* mutant plants could be soil-grown and could flower. Nevertheless, *fsd2-1* mutant plants exhibited extensive leaf bleaching, slow growth, and reduced stature, particularly during the early stages of growth. As *fsd2-1* mutant plants approached flowering, the leaves became greener and plant growth improved although never approaching the size of wild-type plants. The pronounced phenotypes observed with the *fsd2-1* mutant correlated with increased superoxide production that was not observed in the *fsd1-1* mutant, suggesting that FSD1 does not contribute substantially to superoxide reduction under normal growth conditions. Our data show for the first time that the lower chlorophyll levels present in *fsd2-1* mutant plants was accompanied by a substantially lower rate of CO_2_ assimilation, indicating that photosynthetic activity in the mutant is severely compromised. This finding is consistent with our observation that the *fsd2-1* mutant experienced a greater level of damage to PSII as revealed by measuring quantum efficiency under conditions of high light when protein synthesis in the chloroplast was inhibited by chloramphenicol.

The low photosynthetic activity in *fsd2-1* mutant plants was also indicated by a low PSII efficiency, a measure of the proportion of light entering PSII actually used for photochemistry. The reduction in PSII efficiency in *fsd2-1* mutant plants was accompanied by an elevation in total NPQ which differs from the reduction in NPQ previously reported for this mutant [11]. The reason for this difference is unknown although endpoint measurements of NPQ as reported in [11] are highly dependent on the timing of measurements and the conditions employed which were not described in detail in this previous study. The approach employed in the present study used kinetic analysis of the induction of NPQ, PSII efficiency, and Fv/Fm instead of single point measurements. Because each of these parameters can change substantially as a plant responds to light, kinetic analysis reveals considerably more information than single point measurements. Interestingly, most of the increase in NPQ observed in *fsd2-1* mutant in the present study was due to rapidly relaxing components of NPQ such as qE. As photochemical and non-photochemical quenching are competitive processes, this supports the notion that the reduction in photosynthetic activity may be responsible for the increase in rapidly reversible component of NPQ.

FSD2 and FSD3 form heterocomplexes and are present in chloroplast nucleoids [11]. This interaction, however, did not result in increased SOD activity [11], suggesting that the purpose of their interaction appears unrelated to regulating SOD activity. Given that FSD2 and FSD3 co-localize to the same region within the chloroplast, however, might suggest that loss of expression of one of these FeSODs may be compensated by the overexpression of the other.

Overexpression of FSD2 and FSD3 together in Arabidopsis was reported to increase tolerance to oxidative stress imposed by methyl viologen more than did overexpression of FSD2 or FSD3 alone [11]. Our data show for the first time that no decrease in superoxide production was observed in Arabidopsis overexpressing FSD2 or FSD3, either separately or together (Table 1). Moreover, no significant change in chlorophyll levels (Table 2), NPQ or ΦPSII (Fig 2), or the rate of CO_2_ assimilation (Table 3) was observed, suggesting that overexpression of FSD2 and/or FSD3 does not significantly alter photosynthetic parameters under normal growth conditions. Whether FSD2 and/or FSD3 overexpression can confer greater tolerance to natural conditions that result in a greater oxidative load, e.g., under conditions of stress, will be useful to examine in future work.

Myouga et al., 2008 [11] did not investigate whether FSD2 or FSD3 can cross-complement in the absence of expression from the other. Overexpression of FSD2 in the *fsd3-1* mutant failed to compensate for the loss of FSD3 expression in this mutant, at least at the seedling stage. As *fsd3-1* mutant seedlings overexpressing FSD2 failed to survive beyond the seedling stage, it was not possible to determine whether overexpression of FSD2 might compensate for the loss of FSD3 expression at later stages of growth. In contrast to these findings, overexpression of FSD3 in the *fsd2-1* mutant did partially compensate for the loss of FSD2 expression which was readily observable in seedlings and in adult plants which were greener and larger (Fig 5). The overexpression of FSD3 in the *fsd2-1* mutant reduced the level of superoxide production and increased chlorophyll content. Overexpression of FSD3 in the *fsd2-1* mutant also partially reversed the aberrantly higher induction of NPQ during exposure to light.

## Conclusions

The fact that overexpression of FSD3 in the *fsd2-1* mutant only partially compensated for the loss of FSD2 expression and that overexpression of FSD2 in the *fsd3-1* mutant failed to compensate for the loss of FSD3 expression does suggest, however, that FSD2 and FSD3 are functionally distinct to some extent and that the effects that reductions in the level of one FeSOD has on photosynthetic processes cannot be reversed simply by increasing the expression of the other. This suggests that it is not necessarily the additive effect of chloroplast-localized SOD activities that is critical in maintaining plant health under growth conditions. Rather, FSD2 and FSD3 (and possibly CSD2) are likely performing specific functions not easily replaced by the other FeSOD. Such specific functions may involve their ability to interact with other chloroplast components to facilitate their efficient reduction of superoxide radicals or to prevent superoxide radical generation from occurring in the first place. For example, an interaction with catalase or a peroxidase may serve to couple the two step detoxification of superoxide radicals to water thereby conferring greater tolerance to ROS-generating processes. Other interactions with chloroplast components not directly involved in ROS detoxification may serve to localize FSD2 and/or FSD3 to a specific location within the chloroplast that positions it close to sites of superoxide production such as the thylakoid membrane. This might promote superoxide scavenging before the radical has a chance to react with and damage important chloroplast components. It also possible that such positioning close to the thylakoid membrane is necessary for reducing superoxide production in the first place during photosynthesis. Alternatively, the demonstrated interaction between FSD2 and FSD3 [11] may be necessary for their efficient function in the chloroplast in a manner in which over expression of one cannot compensate for loss of expression of the other. A recent finding has shown that LOW SULPHUR UPREGULATED 1 (LSU1) protein physically interacts with FSD2 and activates its enzymatic activity, although whether FSD3 activity is similarly stimulated could not be addressed [30]. Activation of FSDs through interaction with specific partners, however, could be part of the basis of why overexpression of FSD2 or FSD3 cannot complement loss of expression of the other.
